# Supplementary material for: Availability of Guanitoxin in Water Samples Containing Sphaerospermopsis torques-reginae Cells Submitted to Dissolution Tests
Source: Pharmaceuticals (Basel). 2020 Nov 19;13(11):402. doi: 10.3390/ph13110402 (PMC7699232; doi:10.3390/ph13110402)
Supplement: Supplementary file 1 [file pharmaceuticals-13-00402-s001.pdf]

pharmaceuticals

Article

Availability of Guanitoxin in water samples containing *Sphaerospermopsis torques-reginae* cells submitted to dissolution tests

Kelly Afonsina Fernandes [1], Humberto Gomes Ferraz [2,*], Fanny Vereau [2] and Ernani Pinto [1,3,*]

[1] Department of Clinical and Toxicological Analyses, Faculty of Pharmaceutical Sciences, University of São Paulo, Av. Prof. Lineu Prestes, 580, Butantã, CEP 05508-900, São Paulo, SP, Brazil; kelly.af@usp.br
[2] Department of Pharmacy, Faculty of Pharmaceutical Sciences, University of São Paulo, Av. Prof. Lineu Prestes, 580, Butantã, CEP 05508-900, São Paulo, SP, Brazil, sferraz@usp.br; fyvereau@usp.br
[3] Centre of Nuclear Energy in Agriculture, University of São Paulo, Av. Centenário, CEP 303 13416-000, Piracicaba, SP, Brazil
* Correspondence: sferraz@usp.br (H.G.F.); ernani@usp.br (E.P.)

Table S1. Mean and standard deviation (SD) for cell samples of the ITEP-24 strain submitted to dissolution tests (simulated gastric with and without pepsin enzyme solutions). The absolute values presented in the table were obtained through analysis by LC-QqQ-MS/MS.

Minutes	Simulated Gastric Fluid + Pepsin			Simulated Gastric Fluid		
	Mean	SD	N	Mean	SD	N
5	14144	3987	3	30703	875	3
10	23908	1860	3	30511	196	3
15	26620	497	3	29223	505	3
20	27741	151	3	26757	546	3
30	28305	277	3	21090	308	3
45	28679	101	3	19792	600	3
60	29250	70	3	19420	260	3
90	29478	79	3	20962	162	3
120	29972	80	3	20423	70	3

Table S2. Mean and standard deviation (SD) for cell samples of the ITEP-24 strain submitted to dissolution tests (simulated intestinal with and without pancreatin enzyme solutions). The absolute values presented in the table were obtained through analysis by LC-QqQ-MS/MS.

Minutes	Simulated Intestinal Fluid + Pancreatin			Simulated Intestinal Fluid		
	Mean	SD	N	Mean	SD	N
5	1868	710	3	2467	27	3
10	9382	693	3	3307	164	3
15	8103	1326	3	3276	41	3
20	3054	332	3	3393	52	3
30	2921	138	3	3356	82	3
45	3598	61	3	3445	184	3
60	4329	86	3	3519	105	3
90	4912	124	3	3854	146	3
120	5025	40	3	3811	192	3
